# Evaluation of the classification performance and cost-effectiveness of classification criteria in children with systemic lupus erythematosus

**DOI:** 10.3389/fped.2026.1745252

**Published:** 2026-01-30

**Authors:** Ana Luisa Rodríguez-Lozano, Alejandro Gabriel González-Garay, Miguel Ángel Villasis-Keever, Ruth G. Nájera-Velázquez, Marimar Sáez-de-Ocariz, Selma Cecilia Scheffler-Mendoza, Francisco Eduardo Rivas-Larrauri, Marco Antonio Yamazaki-Nakashimada, Silvestre García-De La Puente

**Affiliations:** 1 Immunology Department, Instituto Nacional de Pediatría, Mexico City, Mexico; 2 Department of Research Methodology, Instituto Nacional de Pediatría, Mexico City, Mexico; 3 Pediatric Department, UMAE Hospital de Pediatría “Dr. Silvestre Frenk Freund”, Centro Médico Nacional Siglo XXI, IMSS, Mexico City, Mexico; 4 Dermatology Department, Instituto Nacional de Pediatría, Mexico City, Mexico

**Keywords:** ACR/EULAR-2019, ACR-1997, classification criteria, economic evaluation, SLICC-2012, systemic lupus erythematosus, childhood-onset systemic lupus erythematosus

## Abstract

**Background:**

Childhood-onset systemic lupus erythematosus (cSLE) is a chronic autoimmune disease. In Latin America, the utility of the existing lupus classification criteria and their economic implications remain unexplored.

**Objective:**

To evaluate and compare the utility and cost-effectiveness of the ACR-1997, SLICC-2012, and ACR/EULAR-2019 classification criteria in children with suspected cSLE.

**Methods:**

We prospectively included subjects aged ≤17 who presented to the Immunology Department with suspected cSLE and consented to participate in the study. Exclusion criteria were pregnancy, tuberculosis, immunosuppressive therapy, malignancies, or insufficient data to apply the classification criteria. The ACR-1997, SLICC-2012, and ACR/EULAR-2019 lupus criteria were applied, followed by a referral for expert consensus diagnosis, which served as the standard of reference. We calculated the classification performance (how well a set of classification criteria assigns an individual to SLE or no-cSLE, measuring sensitivity, specificity, among others), direct healthcare costs for each classification, and developed a decision tree and quadrant graph for economic evaluation.

**Results:**

Ninety-six subjects (83% female) with a median age of 13.5 years were included in this study. The expert consensus diagnosis identified cSLE in 43 subjects; ACR and ACR/EULAR each identified 42 cases, while SLICC identified 56. The sensitivity was 98% for SLICC-2012, 84% for ACR-1997, and ACR/EULAR-2019, *p* = 0.000). The cost per subject per classification was USD 287.62 for SLICC-2012, USD 174.97 for ACR-1997, and USD 310.36 for ACR/EULAR-2019 (*p* = 0.596).

**Conclusion:**

The SLICC-2012 criteria were the most sensitive for the classification of SLE, but not the most cost-effective. ACR-1997 remains the most cost-effective tool in specialized settings; however, the higher sensitivity of SLICC-2012 supports its use to improve early referral.

## Introduction

Systemic lupus erythematosus (SLE) is a chronic autoimmune disease characterized by the production of autoantibodies that affect multiple organs or systems, potentially leading to severe organ damage or death (1, 2). When diagnosed before the age of 18, it is known as childhood-onset systemic lupus erythematosus (cSLE) and accounts for 15%–20% of all SLE cases (3). Given its low annual incidence (0.28–0.9 per 100,000 children), cSLE is considered a rare disease (4).

Due to its clinical heterogeneity, the diagnosis of cSLE often proves challenging, and clinicians are regularly aided by the classification criteria, even though this is not their aim (5). The lupus classification criteria are standardized operational definition of “classified cSLE” and structured tools that may support medical reasoning or referral pathways.

The ACR-1997 classification criteria require patients to meet at least four out of eleven criteria (6). In the pediatric population, Ma et al. (7) reported a sensitivity of 87.2% and a specificity of 100%. Petri et al. proposed the SLICC-2012 classification criteria, which require four out of 17 criteria, but that at least one clinical and one immunologic criterion must be present or that biopsy-proven lupus nephritis must coexist with antinuclear antibodies (ANA) or anti-double-stranded DNA (dsDNA) antibodies. This classification has a sensitivity of 97% and a specificity of 84% (8). Recently, Hartman et al. (9) conducted a systematic review that included four pediatric studies comparing the ACR-1997 and SLICC-2012 classification criteria and concluded that while the SLICC-2012 criteria may classify patients earlier than ACR-1997, the latter may be preferable because of fewer false positives. However, all included studies presented a high risk of bias related to patient selection and study design.

The introduction of the ACR/EULAR classification criteria in 2019 reported sensitivities and specificities of 96% and 93%, respectively. Because autoantibodies mediate SLE, these criteria require a positive ANA test as an entry criterion and incorporate a weighted scoring system based on organ involvement and autoantibody profiles; classifying a patient requires at least 10 points (10).

Recent studies reported an average diagnostic delay of 25 months for the diagnosis of adults with lupus (11). Early diagnosis (≤ 6 months) is associated with fewer relapses, reduced organ damage, and lower healthcare utilization and costs (12, 13). Hussain and Li corroborated these findings in pediatric patients, which are particularly relevant. The pediatric population with lupus faces longer disease trajectories and greater lifetime impacts due to their economic repercussions (14, 15). A meta-analysis, also conducted in subjects with cSLE regarding the diagnostic accuracy for cSLE of ACR-1997, SLICC-2012, and ACR/EULAR-2019, revealed that SLICC has the highest sensitivity, ACR has the highest specificity, and that ACR/EULAR does not outperform the other two criteria (16).

Accurate classification contributes to timely diagnosis, appropriate treatment initiation, better disease control, and reduced complications and costs. Therefore, the objective of this study was to evaluate the classification performance and cost-effectiveness of the ACR-1997, SLICC-2012, and ACR/EULAR-2019 classification criteria in Mexican children with suspected cSLE.

## Materials and methods

We conducted a prospective study that included consecutive subjects under 17 years of age of either sex with suspected cSLE who signed consent and assent. Subjects were admitted to the Immunology Department of the Instituto Nacional de Pediatría (INP, a National Institute of Health - tertiary reference center for autoimmune diseases in Mexico) between March 2018 and April 2022. Subjects with suspicion of cSLE were to have two or more of the following: photosensitivity, telogen effluvium, malar or facial rash, oral or nasal ulcers, vasculitic skin lesions, arthralgia or arthritis, seizures, psychosis, mono- or polyneuritis, hematuria, proteinuria, cytopenias, pleural or pericardial effusion, elevated liver enzymes or creatinine, Raynaud's phenomenon, or positive autoantibodies (ANA, dsDNA, antiphospholipid antibodies, a positive Coombs test, or hypocomplementemia).

Participants with complications at admission that prevented accurate cost attribution, those receiving immunosuppressive therapy, those who were pregnant or had neonatal lupus, those with tuberculosis, malignancies, or lymphoproliferative syndromes, those with known immunodeficiencies, or those who did not have the pertinent information to apply the lupus classification criteria (ACR-1997, SLICC-2012, ACR/EULAR-2019), or previously diagnosed with a rheumatic disease were excluded.

Two observers (SCSM and FERL) were standardized in a training session on the lupus classification criteria. We assessed interobserver reproducibility using the kappa consistency index in a pilot sample of 10 medical records from patients with autoimmune diseases. The pilot sample consists of three patients with childhood-onset systemic lupus erythematosus, three patients with juvenile idiopathic arthritis, two patients with juvenile dermatomyositis, and two patients with IgA vasculitis. The interobserver agreement analysis revealed a kappa index of 0.86 between the two standardized observers. All subjects with suspected cSLE underwent the following protocol: (1) signature of informed consent and assent; (2) clinical evaluation; (3) laboratory tests (complete blood count, urinalysis, complement levels, ANA, dsDNA, anti-Sm, antiphospholipid antibodies, and direct Coombs test); and (4) specialty evaluations based on clinical manifestations. We gathered all the information requested at inclusion and applied the classification criteria (ACR-1997, SLICC-2012, and ACR/EULAR-2019).

After applying the lupus classification criteria, all subjects were assessed by an expert consensus diagnosis, which confirmed cSLE or non-cSLE. Each expert independently and blindly reviewed the patient records and reached a consensus diagnosis, either cSLE or non-cSLE, without knowing the results of applying the lupus classification criteria. The expert consensus diagnosis was formed by nine pediatric subspecialists from the INP, including two nephrologists, two neurologists, one dermatologist, one immunologist, one rheumatologist, and two cardiologists. If the agreement was less than 80% (7/9), the decision about the diagnosis was made by the pediatric rheumatologist. All the specialists had more than 7 years of experience assessing children with SLE.

We conducted an analysis on the cost-effectiveness of the application of the classification criteria from the healthcare system perspective, considering only direct costs such as consultations, laboratory and imaging tests, and specialty evaluations. Costs were calculated via the INP 2023 recovery fee tabulation, which includes the real cost without subsidies. A decision tree model was constructed with 38 decision nodes: 11 for ACR-1997, 17 for SLICC-2012, and 10 for ACR/EULAR-2019. A decision tree is a tree-shaped graphical representation of a step-by-step decision-making process. It begins with an initial point (a rectangle) that poses a question or condition and branches into possible dichotomous responses that may depend on the probability of occurrence (circle) or on an evidence-based decision (diamond). It continues in this way until a final decision (triangle) indicates the probability and cost of each option. This information is used to calculate the incremental cost-effectiveness ratio, which is represented in the quadrant graph, visually comparing the cost-effectiveness of the lupus classification criteria.

We calculated the incremental cost-effectiveness ratio and represented it in a quadrant graph.

At the Immunology Department, we registered 1,455 patients in 2018, with an estimated cSLE prevalence of 14%. According to Bujang et al. (17) who used a formula for comparing two weighted proportions and assuming an 80% power, 5% significance, and sensitivities of 0.80 for ACR-1997 and 0.90 for SLICC-2012, we calculated a required sample size of 104 subjects.

### Ethics approval and consent to participate

We obtained the approval of the IRB and EC, with the registration number: 2018/032. Research and Ethics Committees are officially registered at the Office for Human Research Protections of the NIH (http://ohrp.cit.nih.gov/search/search.aspx) with numbers IRB00008064 and IRB00008065. The approval letter is available upon request. All subjects' information was handled confidentially. Additionally, there is no personally identifiable information in the data presented herein.

### Statistical analysis

We analyzed observer agreement via the kappa index and calculated the median, minimum, and maximum according to a non-normal distribution. We reported categorical variables as frequencies and proportions. We used Pearson's chi-square test or Fisher's exact test to compare categorical variables between cSLE and non-cSLE, while the Mann–Whitney *U*-test was used for continuous variables, with a significance threshold of *p* ≤ 0.05. We calculate the sensitivity, specificity, predictive values, and ROC curves for the ACR-1997, SLICC-2012, and ACR/EULAR-2019 classification criteria using expert consensus diagnosis as the gold standard. To compare the sensitivity and specificity between the three classification criteria, we used McNemar's test. We performed all the analyses using STATA 18.1. Finally, to calculate the costs of applying the classification criteria, we used the INP recovery fee tabulation, which presents the real cost of supplies. The decision tree was constructed using Tree Age Pro software to evaluate cost-effectiveness.

## Results

A total of 105 subjects with suspected cSLE were evaluated; two declined to participate and seven were excluded for incomplete screening, leaving 96 patients who met the selection criteria.

Among the 96 participants, 80 (83%) were female and 16 (17%) were male (*p* < 0.001). The median age was 13 years and 6 months, ranging from 3 years and 2 months to 17 years and 9 months.

After completing the clinical evaluations and obtaining laboratory results, the ACR-1997, SLICC-2012, and ACR/EULAR-2019 classification criteria were applied. These criteria identified cSLE in 42 (43.7%), 56 (58.3%), and 42 (43.7%) participants, respectively. The expert consensus diagnosis identified 43 subjects with cSLE (45%) and non-cSLE in 53 (55%) participants (Table 1). Supplementary Table S1 shows the diagnoses of the participants in the non-cSLE group.

**Table 1:** Comparison between expert consensus diagnosis and lupus classification criteria. *N* = 96.

Classification criteria	Expert consensus diagnosis		Total
	cSLE	Non-cSLE	
ACR – 1997 positive	42 (97.6%)	0	42
ACR – 1997 negative	1 (2.4%)	53 (100%)	54
SLICC – 2012 positive	43 (100%)	13 (24.5%)	56
SLICC – 2012 negative	0	40 (75.5%)	40
ACR/EULAR – 2019 positive	42 (97.6%)	0	42
ACR/EULAR – 2019 negative	1 (2.4%)	53 (100%)	54
Total	43	53	96

For each classification criteria, there are two options: positive if the subjects met the classification criteria and negative if they did not. For example, “ACR-1997 positive” represents the subjects who met at least four out of 11 criteria; “ACR-1997 negative” represents those who did not meet the ACR classification criteria.

The median age of patients diagnosed with cSLE was 14 years (7.41–17.75), whereas in the non-cSLE group, it was 12 years (3.16–17) (*P* = 0.013). Among patients with cSLE, 37 (86%) were female, whereas 43 (81%) were in the non-cSLE group (*p* = 0.521).

We observed laboratory differences between the cSLE and non-cSLE groups—particularly in hemoglobin levels, platelet and lymphocyte counts, and serum creatinine and complement levels—all of which were lower in the cSLE subjects (see Table 2).

**Table 2:** Features of the subjects with cSLE and non-cSLE based on the expert consensus diagnosis.

Features of the subjects	cSLE *n* = 43 Median (min – max)	Non-cSLE *n* = 53 Median (min – max)	*P*
Age, years	14.20 (7.41–17.75)	12.24 (3.16–17)	0.013*
Proteinuria (g/d)	0.27 (0–11)	0.03 (0–6.6)	0.002*
Hb (mg/dl)	11.1 (5.4–15.9)	14.2 (6.9–16.5)	0.000*
Lymphocytes (mm^3^)	1,200 (200–4,400)	1,900 (400–5,500)	0.005*
Platelets (×1,000/mm^3^)	217 (6–534)	276 (39–700)	0.015*
C3 (mg/dl)	80.5 (18.9–147)	117 (53.4–189)	0.000*
C4 (mg/dl)	8.5 (0.6–43)	19.5 (6.68–41)	0.000*
ACR-1997 criteria (frequency)	6 (2–9)	2 (0–5)	0.000*
SLICC-2012 criteria (frequency)	8 (3–13)	3 (0–5)	0.000*
ACR/EULAR-2019 criteria (score)	21 (4–37)	8 (0–27)	0.000*

Statistical test: Mann–Whitney *U*-test. C3, C4: Complement fractions C3 and C4. ACR-1997 and SLICC-2012 require four or more criteria for classification. ACR/EULAR-2019 classifies subjects with ANA+ and 10 or more points.

* *P* ≤ 0.05.

With respect to antiphospholipid antibodies, 26 (61%) subjects in the cSLE group tested positive, whereas 10 (21%) were in the non-cSLE group. We observed a positive direct Coombs test in 29 (74%) subjects in the cSLE group and in 5 (16%) participants in the non-cSLE group see Table 3.

**Table 3:** Characteristics of the sample and classification criteria based on the expert consensus diagnosis.

Features of the subjects	cSLE *n* = 43 Frequency (%)	Non-cSLE *n* = 53 Frequency (%)	*P*
Sex (female)	37 (86)	43 (81)	0.521
Malar rash	18 (42)	13 (24)	0.071
Mucosal ulcers	16 (37)	8 (15)	0.013*
Synovitis			
ACR-1997	25 (58)	19 (36)	0.029*
SLICC-2012	25 (58)	20 (38)	0.046*
ACR/EULAR-2019	25 (58)	18 (43)	0.048*
Neurologic			
ACR-1997	3 (7)	5 (9)	0.665
SLICC-2012	5 (12)	9 (17)	0.460
ACR/EULAR-2019	3 (7)	6 (12)	0.470
Nephritis			
ACR-1997	18 (43)	9 (17)	0.005*
SLICC-2012	19 (44)	12 (23)	0.025*
ACR/EULAR-2019	20 (48)	10 (19)	0.001*
Serositis	8 (19)	2 (4)	0.018*
Immune cytopenias	33 (77)	19 (36)	0.000*
Hemolytic anemia	18 (42)	3 (6)	0.000*
ANA +	39 (91)	20 (38)	0.000*
dsDNA +	17 (41)	0 (-)	0.000*
Anti-Sm	16 (42)	0 (-)	0.000*
APS +	26 (61)	10 (21)	0.000*
Direct Coombs test +	29 (74)	5 (16)	0.000*

Statistical test: Pearson's chi-square test. ANA, antinuclear antibodies; dsDNA, double-stranded anti-deoxyribonucleic acid antibodies; Anti-Sm, anti-Smith; APS, antiphospholipid antibodies.

* *P* ≤ 0.05.

### Classification test analysis

The SLICC-2012 criteria demonstrated the highest sensitivity for classification of 98%, whereas ACR-1997 and ACR/EULAR-2019 each showed a sensitivity for classification of 84% (*p* = 0.000). The specificity for classification was 0.735 for SLICC-2012 and 0.886 for both ACR-1997 and ACR/EULAR-2019 (*p* = 0.007). While the ACR-1997 and ACR/EULAR-2019 classification criteria both had one false-negative case, SLICC-2012 had no false negatives; however, it had thirteen false-positive cases. The area under the receiver operating characteristic curve (AUC) did not differ significantly between the classifications (*p* = 0.897). The detailed classification performance metrics are presented in Table 4.

**Table 4:** Classification test performance: comparison of lupus classification criteria and expert consensus diagnosis as reference standard.

Diagnosis test analysis	ACR-1997 (IC95%)	SLICC-2012 (IC95%)	ACR/EULAR-2019	*P*
Sensitivity	0.837 (0.763–0.911)	0.976 (0.945–1.000)	0.837 (0.763–0.911)	0.000*
Specificity	0.886 (0.822–0.950)	0.735 (0.647–0.823)	0.886 (0.822–0.950)	0.007*
PPV	0.857 (0.800–0.916)	0.750 (0.589–0.940)	0.857 (0.800–0.916)	0.061
NPV	0.870 (0.813–0.919)	0.975 (0.914–1.038)	0.870 (0.813–0.919)	0.006*
Accuracy	0.864 (0.807–0.923)	0.843 (0.787–0.901)	0.864 (0.807–0.923)	0.681
AUC	0.862 (0.781–0.943)	0.856 (0.777–0.935)	0.862 (0.781–0.943)	0.897

PPV, positive predictive value; NPV, negative predictive value; AUC, area under the curve. Statistical test: Chi-square.

* *P* ≤ 0.05.

### Comparative tests between classification criteria

We performed the analysis focusing specifically on the discordant cases, ignoring the concordant cases. McNemar's test allowed us to corroborate the statistically significant difference, mainly in the specificity of the tests, as shown below.

#### Sensitivity

Among Expert Consensus Diagnosis- positive subjects (*n* = 43), SLICC-2012 classified 43/43 as positive, and ACR-1997 and ACR/EULAR-2019 42/43; sensitivity 0.976; McNemar's test showed no significant difference (exact *p* = 1.0).

Among Expert Consensus Diagnosis- positive subjects (*n* = 43), ACR/EULAR-2019 and ACR-1997 both classified 42/42 as positive. Sensitivity 1.0; McNemar's test *p* = 1.00.

#### Specificity

Among Expert Consensus Diagnosis- negative subjects (*n* = 53), SLICC-2012 classified 40/53 and ACR-1997 and ACR/EULAR-2019 53/53; specificity 1, McNemar's test *p* = 0.0002.

Among Expert Consensus Diagnosis- negative subjects (*n* = 53), ACR/EULAR-2019 and ACR-1997 showed identical specificity (no discordant pairs; McNemar's test *p* = 1.0).

### Cost-effectiveness estimation

**Figure 1:**
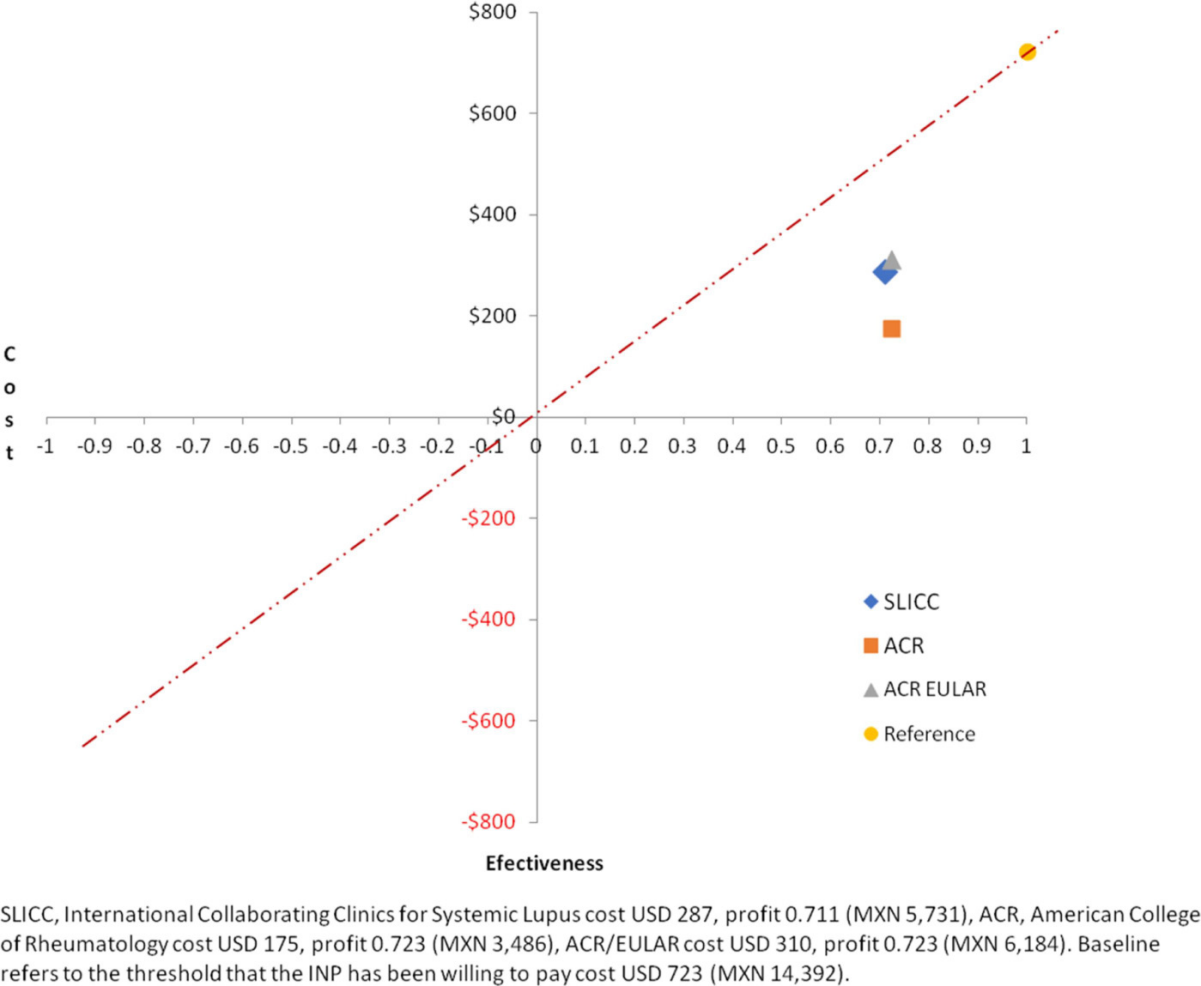
Cost-effectiveness of the ACR-1997, SLICC-2012, and ACR/EULAR-2019 classification criteria.

We built a decision tree for economic analysis with three branches: ACR-1997, SLICC-2012, and ACR/EULAR-2019. Each branch splits by item count, then further by probability nodes based on clinical and auxiliary diagnostic tests. Each pathway ends at a cost-effectiveness outcome (Supplementary Figure S1). This model allows for calculation and visualization of cost-effectiveness on a quadrant plot. All three criteria are located in the upper right quadrant, indicating effectiveness; however, they differ in cost (Table 5, Figure 1).

**Table 5:** Cost-effectiveness analysis.

Costs and classification performance	ACR-1997 Mean	SLICC-2012 Mean	ACR/EULAR-2019 Mean	*P*
Cost per subject USD	174.97	287.62	310.36	0.041*
	Pb (IC95%)	Pb (IC 95%)	Pb (IC95%)	
Diagnosis test analysis	0.724	0.711	0.724	0.066**
AUC	0.862 (0.791–0.932)	0.835 (0.766–0.904)	0.862 (0.791–0.932)	0.789***

* Decision tree cost analysis with Monte Carlo test.

** X2 diagnostic test.

*** AUC, area under the curve. Test for equality of areas under the curve.

Bold value indicates *P* ≤ 0.05.

### Incremental cost

Upon folding the decision tree, we observed a total (96 subjects) potential savings via ACR-1997 of USD 10,752 (MX$ 183,312.00) compared with SLICC-2012 or USD 12,960 (MX$ 220,310.40) with ACR/EULAR-2019.

## Discussion

To the best of our knowledge, this study is the first to conduct an economic evaluation of the SLICC-2012, ACR-1997, and ACR/EULAR-2019 classification criteria in children, focusing on the costs and performance associated with applying each criteria set. Importantly, classification criteria are primarily intended to define homogeneous groups for research. Therefore, throughout this discussion, we interpret our findings as the comparative value of these criteria as standardized operational definitions of “classified cSLE” and as structured tools that may support clinical reasoning or referral pathways; however, they do not replace clinical diagnosis in routine practice. Accordingly, our economic analysis estimates the cost of applying each criteria set to classify patients, not the cost of diagnosing SLE.

We found that while all three classification criteria demonstrated broadly similar classification performance (sensitivity, specificity, accuracy, and AUC), against expert consensus diagnosis in our sample, SLICC-2012 and ACR/EULAR-2019 were more costly to apply than ACR-1997. The higher sensitivity for classification observed with SLICC-2012 is biologically and clinically plausible given its broader definition of cutaneous and neurologic involvement, hemolytic anemia, inclusion of complement levels, separate accounting of autoantibodies, and the possibility of classification via renal biopsy. These features likely contribute to earlier or more inclusive classification when compared with ACR-1997 or ACR/EULAR-2019, consistent with prior reports (16).

Our findings align with previous reports, showing that SLICC-2012 had higher sensitivity for classification but lower specificity than the ACR-1997 or ACR/EULAR-2019 criteria (18). Hartman et al. suggested that ACR-1997 may reduce false positives compared with SLICC-2012 (9). In our study, 13 subjects from the non-SLE sample were initially identified by the SLICC-2012 but not by the expert consensus diagnosis. These cases may fall under the category of “incomplete lupus”, characterized by positive ANA and some clinical features or a history of autoimmunity without meeting the minimum classification criteria (19). Conversely, we also observed that 20 participants failed to meet the ACR/EULAR-2019 criteria because they tested negative for ANA, reflecting a key structural aspect of that criteria set (ANA as an entry criterion) that may prevent classification even when clinical and immunologic features are otherwise substantial. Notably, these individuals were followed longitudinally in the outpatient clinic, reinforcing the point that classification criteria may not fully capture the complexity of diagnostic decision-making in real-world care.

Although Chang's meta-analysis reported good sensitivity for ACR/EULAR-2019, it did not outperform SLICC-2012 in terms of sensitivity or ACR-1997 in terms of specificity (16) In our experience, the three classification criteria had comparable overall accuracy (*P* = 0.681). More recently, Babgi's retrospective study compared the performance of SLICC-2012 and ACR/EULAR-2019 for early detection of cSLE. Their results differ from ours mainly because they find the EULAR/ACR-2019 classification criteria slightly more sensitive than SLICC, and ACR/EULAR-2019 having a higher rate of false positives, but, as in our case, with no statistically significant differences (20).

This distinction between classification and diagnosis is critical when interpreting the implications of our findings. A recent study in adults reported discordance between clinician diagnosis and ACR/EULAR-2019 classification, concluding that ACR/EULAR-2019 performs better as a classification tool than as a diagnostic tool. Together with our observations, this supports the view that classification criteria should not be presented as diagnostic standards in clinical practice; rather, they can be understood as structured frameworks that may aid recognition of probable disease, standardize reporting, or define operational endpoints such as incident “classified SLE” in research settings.

Despite its good sensitivity and specificity, the ACR-1997 has faced criticism. For instance, Petri and Magder argued that the tool overemphasizes cutaneous criteria—four out of 11—while more severe organ systems such as the kidneys or the central nervous system, are underrepresented. To address these limitations, Petri and colleagues developed the SLICC-2012 classification criteria (8). Pons–Estel et al. compared ACR-1997 and SLICC-2012 in the GLADEL cohort and reported that the SLICC-2012 outperformed the diagnosis of an experienced physician (21–23). Among the 850 subject classified by either classification criteria, 254 were identified earlier by the SLICC-2012 and 295 later.

Since SLE lacks pathognomonic features, its diagnosis is often challenging. Accurate diagnosis depends heavily on the physician's expertise and the integration of clinical and laboratory data. Before 1980, diagnostic delays averaged 4 years (23, 24). Although several efforts have aimed to improve early-stage diagnosis of SLE, this remains an unmet need. Using current classification criteria as diagnostic tools has its drawbacks, as described by Aringer et al. (25) Other authors argue that using all three classification criteria ensures the highest sensitivity (26), but at what cost?

Early diagnosis of SLE is crucial for limiting the disease progression, reducing complications, and lowering healthcare costs. Health technology assessment aims to lead clinical and policy decisions by providing reliable information on health outcomes and resource utilization, achieving cost-effective decisions (27). One study using administrative data from two pediatric rheumatology centers reported cumulative care costs of $3,965,048 USD for 119 cSLE patients between 2001 and 2004, approximately $14,944 USD per patient per year (28). This report has been over 20 years, and although there are several economic studies on SLE, the representation of the pediatric population is lacking or not described. These studies predominantly focus on disease evolution and treatment, not on its diagnosis, which also generates healthcare costs (29–35).

Data on the costs associated with applying lupus classification criteria are lacking, making our results particularly valuable. Our quadrant analysis shows that, whereas SLICC-2012 has the highest sensitivity, it is also the most expensive. In healthcare settings without the expertise of pediatric rheumatologists or immunologists, investing in more sensitive tools such as SLICC-2012 may help ensure timely referral to specialized centers. These conditions are particularly relevant for diseases such as lupus, where early aggressive treatment is critical to halting disease progression.

Following Turchetti's methodology (36), we adjusted for inflation in Mexico from January 2022 to February 2025 (17.56%) to facilitate the comparison of our results. The inflation for the period from January 2022 to February 2025 was 17.56% (37). Subsequently, we converted costs from Mexican pesos to US dollars using the exchange rates from January 2023 (38). January 2023 was selected as the start date because it corresponds to the date we calculated the costs from the fee table of the INP. The adjusted cost per subject was MXN 5,731.40 (USD 287.62) for SLICC-2012, MXN 3,486.59 (USD 174.97) for ACR-1997, and MXN 6,184.47 (USD 310.36) for ACR/EULAR-2019.

In Mexico and Latin America, economic constraints and limited access to specialists outside major urban centers often hinder patients from seeking timely medical care. Our findings indicate that the SLICC-2012 and ACR/EULAR-2019 are not more cost-effective than ACR-1997. However, if we consider the long-term costs of complications resulting from delayed diagnosis and acknowledge that the SLICC-2012 classifies up to 15% more patients, it becomes relevant to tailor the choice of classification criteria to the institutional context. As mentioned earlier, the classification criteria may support less experienced physicians in recognizing and suspecting the diagnosis. The Instituto Nacional de Pediatría is a Latin American reference center not only for patients but also for medical training. With an understanding of the economic challenges and lack of specialists in the region, based on our results, we suggest using the ACR-1997 lupus classification criteria in referral centers with experienced staff, as it may be sufficient and offer significant cost savings (up to USD 12,960). In non-specialized settings, the greater sensitivity of SLICC-2012 justifies its use to facilitate early identification and referral.

Despite the implications of the findings, our study has some limitations. Due to the nature of the disease, we decided to apply the expert consensus diagnosis after using the lupus classification criteria, which may lead to an overestimation of expert opinion. Nonetheless, since we consider the expert as the best benchmark, this does not affect our results. Other limitations are the loss of subjects in the initial stages, preventing their evaluation by the expert consensus diagnosis, the limited sample size, and, primarily due to the shift in healthcare delivery caused by the pandemic, we do not consider the costs associated with delayed diagnosis and overdiagnosis The narrow available literature also restricted comparisons with our results. Despite these constraints, the major strength of this study lies in the adjustment of cost indicators (unsubsidized cost, representing the actual cost) and their update for inflation to provide current and accurate information. Additionally, as a referral center, we were able to include newly referred subjects without a definitive diagnosis, and the institution has trained and experienced specialists available to perform the assessments.

## Conclusion

Childhood-onset SLE is a life-threatening condition, and cost-effective diagnostic tools are essential, particularly in middle-income countries such as Mexico. In this study, SLICC-2012 was the most sensitive classification criteria, whereas ACR/EULAR-2019 was the most expensive. Although we did not find statistically significant differences in effectiveness, using ACR-1997 instead of SLICC-2012 or ACR/EULAR-2019 would result in estimated savings of about USD 10,752 (MX$183,312.00) compared with SLICC-2012 or USD 12,960 (MX$220,310.40) with ACR/EULAR-2019. ACR-1997 remains the most cost-effective option in specialized centers; however, in non-specialized settings, SLICC-2012 may be the most suitable for identifying potential cases and facilitating timely referral by non-specialists, given its higher sensitivity.

## Data Availability

The original contributions presented in the study are included in the article/Supplementary Material, further inquiries can be directed to the corresponding author.

## Supplementary material

Supplementary Table S1 Diagnoses present in the non-cSLE sample. *N* = 53.

Supplementary Figure S1 DecisionTreeClassifCrit.jpg – decision tree.
