# Supplementary material for: Evaluation of the classification performance and cost-effectiveness of classification criteria in children with systemic lupus erythematosus
Source: Front Pediatr. 2026 Jan 30;14:1745252. doi: 10.3389/fped.2026.1745252 (PMC12901401; doi:10.3389/fped.2026.1745252)
Supplement: Supplementary Table S1 — Diagnoses present in the non-cSLE sample. N = 53. [file Table1.docx]

**Supplementary Table 1. Diagnoses present in the non-cSLE sample. N = 53**

| Disease | Frequency (%) |
| --- | --- |
| Incomplete cSLE* | 13 (13) |
| IgA vasculitis | 10 (10) |
| Renal disease | 5 (5) |
| Juvenile idiopathic arthritis | 4 (4) |
| Encephalitis** | 4 (4) |
| Juvenile dermatomyositis (JDM) | 3 (3) |
| Tiroiditis | 2 (2) |
| Systemic vasculitis | 2 (2) |
| Neuritis | 2 (2) |
| VEB infection | 2 (2) |
| Immune thrombocytopenia | 1 (1) |
| APS | 1 (1) |
| Overlap syndrome JDM-cSLE | 1 (1) |
| Raynaud Phenomenon | 1 (1) |
| Demyelinating disease | 1 (1) |
| Posterior cord myelopathy | 1 (1) |

Incomplete cSLE: patients who did not meet SLICC, ACR, or ACR/EULAR classification criteria. **Three subjects with anti-NMDAr (N-methyl-D-aspartate receptor) encephalitis and one with a diagnosis of encephalitis. APS: Antiphospholipid syndrome with stroke.
